# The Immunological Effect of Oxygen Carriers on Normothermic *Ex Vivo* Liver Perfusion

**DOI:** 10.3389/fimmu.2022.833243

**Published:** 2022-06-22

**Authors:** Heather Jennings, Kristin N. Carlson, Chris Little, Joshua C. Verhagen, Jeevan Nagendran, Yongjun Liu, Bret Verhoven, Weifeng Zeng, Stacey McMorrow, Peter Chlebeck, David P. Al-Adra

**Affiliations:** ^1^ Division of Transplantation, Department of Surgery, University of Wisconsin School of Medicine and Public Health, Madison, WI, United States; ^2^ Department of Surgery, University of Alberta, Edmonton, AB, Canada; ^3^ Department of Pathology, University of Wisconsin School of Medicine and Public Health, Madison, WI, United States

**Keywords:** liver, transplantation, normothermic perfusion, oxygen carrier, immune system

## Abstract

**Introduction:**

Normothermic *ex vivo* liver perfusion (NEVLP) is an organ preservation method that allows liver graft functional assessment prior to transplantation. One key component of normothermic perfusion solution is an oxygen carrier to provide oxygen to the liver to sustain metabolic activities. Oxygen carriers such as red blood cells (RBCs) or hemoglobin-based oxygen carriers have an unknown effect on the liver-resident immune cells during NEVLP. In this study, we assessed the effects of different oxygen carriers on the phenotype and function of liver-resident immune cells.

**Methods:**

Adult Lewis rat livers underwent NEVLP using three different oxygen carriers: human packed RBCs (pRBCs), rat pRBCs, or Oxyglobin (a synthetic hemoglobin-based oxygen carrier). Hourly perfusate samples were collected for downstream analysis, and livers were digested to isolate immune cells. The concentration of common cytokines was measured in the perfusate, and the immune cells underwent phenotypic characterization with flow cytometry and quantitative reverse transcription polymerase chain reaction (qRT-PCR). The stimulatory function of the liver-resident immune cells was assessed using mixed lymphocyte reactions.

**Results:**

There were no differences in liver function, liver damage, or histology between the three oxygen carriers. qRT-PCR revealed that the gene expression of nuclear factor κ light chain enhancer of activated B cells (NF-kB), Interleukin (IL-1β), C-C motif chemokine ligand 2 (CCL2), C-C motif chemokine ligand 7 (CCL7), and CD14 was significantly upregulated in the human pRBC group compared with that in the naive, whereas the rat pRBC and Oxyglobin groups were not different from that of naive. Flow cytometry demonstrated that the cell surface expression of the immune co-stimulatory protein, CD86, was significantly higher on liver-resident macrophages and plasmacytoid dendritic cells perfused with human pRBC compared to Oxyglobin. Mixed lymphocyte reactions revealed increased allogeneic T-cell proliferation in the human and rat pRBC groups compared to that in the Oxyglobin group.

**Conclusions:**

Liver-resident immune cells are important mediators of rejection after transplantation. In this study, we show that the oxygen carrier used in NEVLP solutions can affect the phenotype of these liver-resident immune cells. The synthetic hemoglobin-based oxygen carrier, Oxyglobin, showed the least amount of liver-resident immune cell activation and the least amount of allogeneic proliferation when compared to human or rat pRBCs. To mitigate liver-resident immune cell activation during NEVLP (and subsequent transplantation), Oxyglobin may be an optimal oxygen carrier.

## Introduction

The rising incidence of chronic liver disease coincides with the rising demand for liver transplantation from a limited pool of organ donors (1, 2). To address this critical organ shortage, clinicians have utilized marginal organs from elderly donors, donation after cardiac death (DCD), and donors with multiple comorbidities, all of which are associated with poorer clinical outcomes in part due to their poor tolerance of the standard preservation method, static cold storage (SCS) (3, 4). In contrast to SCS, normothermic *ex vivo* liver perfusion (NEVLP) is an organ preservation method that maintains the liver at physiologic temperature, thereby preserving metabolic functions. Therefore, NEVLP allows liver graft functional assessment and the opportunity for organ modification prior to transplantation. Clinical studies indicate improved transplant outcomes for organs preserved by machine perfusion (5, 6), and the efficacy of NEVLP has been demonstrated in two randomized clinical trials (7, 8).

To preserve metabolic function during NEVLP, an oxygen carrier, nutrients, and medications are perfused through the liver (9–11). A common NEVLP oxygen carrier is packed red blood cells (pRBCs) (12). However, the use of pRBCs entails certain drawbacks, including hemolysis in centrifugal/rotary pump perfusion systems (13), immune-mediated phenomena, blood-borne pathogen transmission (14), cross-matching difficulties, and pRBC being a limited resource (15). Hemoglobin-based oxygen carriers, such as Hemopure and Oxyglobin (HbO2 Therapeutics), have been presented as a promising alternative to pRBC in *ex vivo* normothermic perfusion procedures (11, 16, 17). Oxyglobin is a polymerized hemoglobin synthesized from bovine RBCs and purified to avoid blood-borne transmissions (16). However, studies comparing pRBC and acellular perfusates lack focused investigation of the immunological effects of various oxygen carriers on the organ being perfused.

Liver-resident immune cells are key players in understanding the development of transplant tolerance or rejection (18, 19). It has been previously shown that NEVLP increases the expression of damage-associated molecular pattern (DAMP) proteins in the liver (20). Furthermore, we found that NEVLP-associated inflammation in the liver affects the phenotype of liver-resident dendritic cells (DCs), which may have undesirable downstream immunological consequences (21). Therefore, reducing the inflammatory environment within the liver during NEVLP for experimental or clinical purposes is of critical importance.

In this study, we performed NEVLP on Lewis rat livers and assessed the immunological effects of three different oxygen carriers by measuring inflammatory cytokine secretion and gene expression signatures. In addition, given the role that liver-resident DCs play in allograft immunogenicity, we specifically evaluated the effect of different oxygen carriers on the phenotype of these cells and assessed their stimulatory function in mixed lymphocyte reactions (MLRs).

## Materials and Methods

### Animals

Male Lewis and Brown Norway rats (Charles River Laboratories, Wilmington, MA, USA) aged 4–15 weeks weighing 320 g ± 11 g [mean ± standard error of the mean (SEM)] were used in all experiments. Animals were housed in specific pathogen-free conditions in animal care facilities at the University of Wisconsin (UW)-Madison in accordance with institutional guidelines. The study protocol was approved by the Institutional Animal Care and Use Committee at the UW-Madison, and all animals were treated ethically.

### Animal Surgery and Liver Procurement

**Figure 1:**
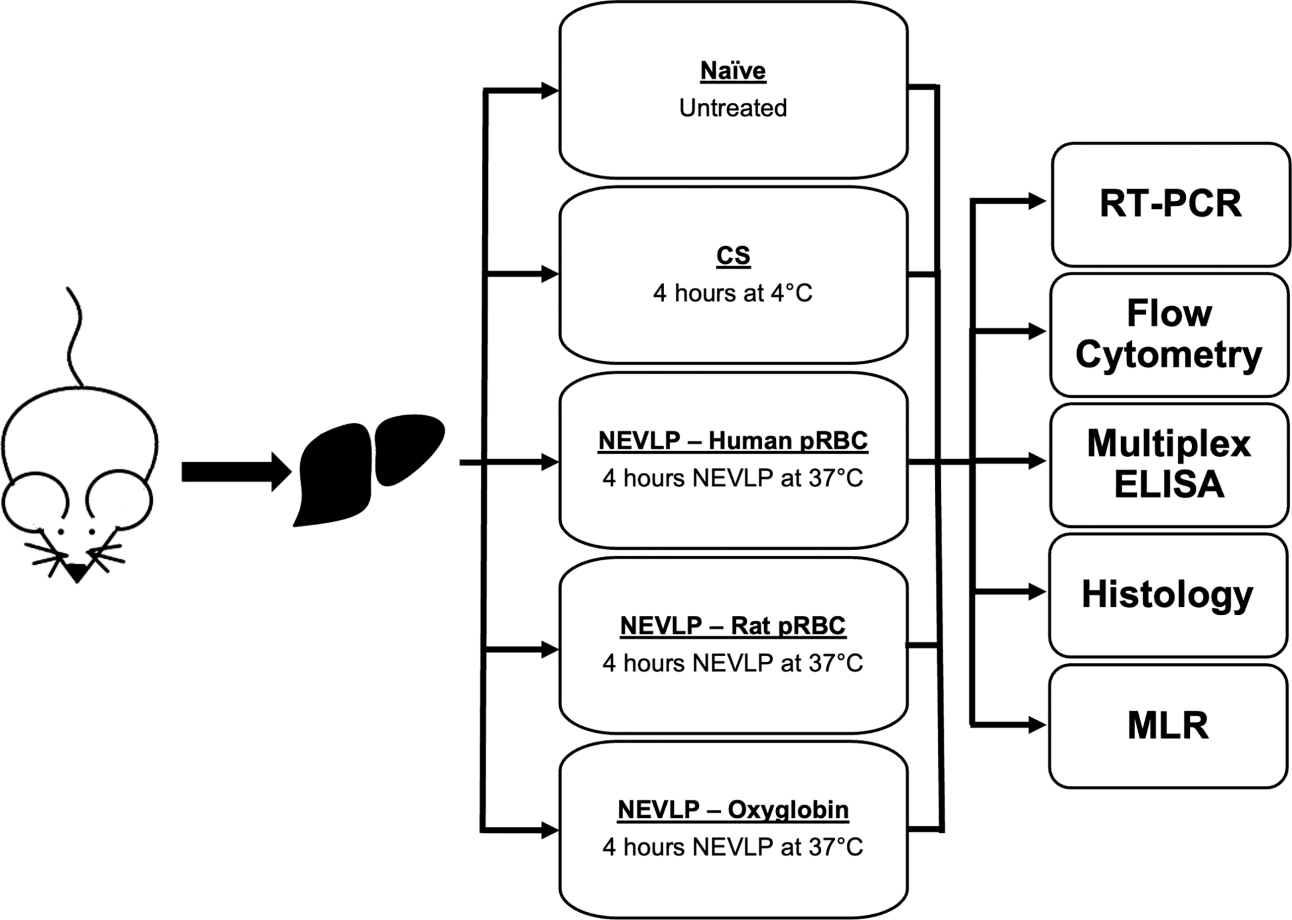
Experimental design. pRBC, packed red blood cells; NEVLP, normothermic ex vivo liver perfusion; CS, Cold storage.

Animals were randomly assigned to one of three treatment groups: naive (n = 4), SCS (n = 4), NEVLP with human pRBC (n = 6), NEVLP with rat pRBC (n = 7), and NEVLP with Oxyglobin (n = 8) (**Figure 1**). Surgeries were performed under inhaled 5% isoflurane (Phoenix, St. Joseph, MO, USA) anesthesia for induction and 2%–3% isoflurane maintenance. After disinfection with Betadine (Purdue Pharma LP, Stamford, CT, USA), the abdominal cavity was opened by a midline and transverse incision and the portal vein was exposed. The common bile duct was cannulated with a 24-gauge angiocatheter (BD Biosciences). The hepatic artery and gastrosplenic and duodenopancreatic branches of the portal vein were isolated and ligated. Heparin (400U, Fresenius Kabi, Lake Zurich, IL, USA) was injected through the inferior vena cava and allowed to circulate for 5 min. The portal vein was then cannulated with a 1.3-mm miniball cannula with basket tip (Harvard Apparatus, Holliston, MA, USA) and flushed with 20 ml of cold 0.9% saline (Baxter). Livers were then explanted and weighed. Naive livers were processed immediately, SCS livers were stored on ice for 4 h and then processed, and NEVLP livers were connected to the perfusion machine with minimal cold ischemic time (<5 min).

### Perfusate Composition

NEVLP perfusate was composed of William’s E Media (Quality Biological, Gaithersburg, MD, USA; 65 ml for pRBC oxygen carriers and 50 ml for Oxyglobin) with the following additives: 3,250U each penicillin/streptomycin (Life Technologies Corporation, Grand Island, NY, USA), 500U heparin (Fresenius Kabi, Lake Zurich, IL, USA), 1 mg insulin (Sigma-Aldrich), 1.25 mg hydrocortisone (Pfizer, New York, NY, USA), and 15 mg papaverine (American Regent, Shirley, NY, USA). In addition, the total perfusate volume of 100 ml contained sodium pyruvate 0.65 mM (Sigma-Aldrich, St. Louis, MO, USA), L- glutamine 1.30 mM (Sigma-Aldrich), and human albumin 1% (Baxter).

The oxygen carriers added to the above perfusate were human pRBC, rat pRBC, or Oxyglobin. Human pRBC (30 ml; American Red Cross, Madison, WI, USA) of blood type A were leukoreduced, irradiated, and used within expiration date. Pooled rat pRBC was collected from the aortic puncture of donor Lewis rats into citrate phosphate dextrose adenine (CPDA) and centrifuged for 10 min at 2,200g. Plasma and buffy coat were discarded, and pRBC was resuspended in AS-5, then drop-filtered through an Acrodisc WBC filter (Pall Gellman), stored at 4°C, and used within 1 week (30 ml). Oxyglobin (46 ml) was kindly provided by HbO2 Therapeutics, LLC (Souderton, PA, USA).

### Machine Perfusion

NEVLP was performed as previously described (17). Briefly, warmed oxygenated perfusate was circulated through the portal vein at 1.8 ml/min/g liver by peristaltic pump. Temperature, pressure, flow rate, and oxygen saturation were monitored throughout perfusion (Hugo Sachs Elektronic, Harvard Apparatus). The oxygenator was supplied by 95% O_2_/5% CO_2_ gas to maintain >95% saturation of the perfusate; the temperature of the liver was maintained at 37°C.

An i-STAT point-of-care analyzer (CG4+ and CHEM8+ cartridges, Abbott Point of Care Inc., Abbott Park, IL, USA) was used for hourly inflow perfusate testing. Samples for qPCR and ELISA were collected from the inflow port and centrifuged for 15 min at 20,000g, and supernatants were stored at -80°C until further processing.

### Liver Damage and Inflammation Assessment

For liver damage analysis, liver-type arginase 1 (ARG1), aspartate transaminase 1 (AST), α-glutathione S-transferase (GSTα), sorbitol dehydrogenase (SDH), and 5′-nucleotidase (5′-NT) were measured in the perfusate by Milliplex™ Rat Liver Injury Panel (Millipore Sigma, Billerica, MA, USA) according to the manufacturer’s recommendations. Data were acquired on the Luminex MAGPIX (Austin, TX, USA) and analyzed in the Belysa software package v1.0.19. For perfusate cytokine analysis, the LEGENDplex™ Rat Inflammation Panel (BioLegend, San Diego, CA, USA) was used to detect IL-1α, IL-1β, IL-6, IL-10, IL-12p70, IL-17A, IL-18, IL-33, CXCL1, CCL2 [Monocyte Chemoattractant Protein-1 (MCP-1)], granulocyte-macrophage colony-stimulating factor (GM-CSF), interferon (IFN-γ), Tumor necrosis factor (TNF-α), according to the manufacturer’s recommendations. Data were acquired on a BD FACS Calibur and analyzed in LEGENDplex™ software package v7.1.21.

### Histopathology and Immunohistochemistry

Prior to liver digestion, left median lobe liver sections were fixed in 4% paraformaldehyde and embedded in paraffin. Slides cut at 4-μm thickness were stained with hematoxylin and eosin (H&E). An expert liver pathologist (YL) blindly scored the severity of histologic damage to the liver according to the Suzuki criteria (21). Representative micrographs were collected at appropriate magnification using an Olympus DP73 equipped microscope (Olympus, Tokyo, Japan).

### Liver-Resident Immune Cell Isolation

Liver-resident immune cells were isolated as previously described (21). Briefly, 100 ml of warmed 1× Hank’s balanced salt solution (HBSS, Worthington, Lakewood, NJ, USA) containing 25 mM N-2-Hydroxyethylpiperazine-N'-2-Ethanesulfonic Acid (HEPES) (Sigma-Aldrich), 4.2 mM NaHCO_3_ (Sigma-Aldrich), and 83 μM Ethylenediaminetetraacetic acid (EDTA) (Sigma- Aldrich) was perfused through the portal vein. Subsequently, 100 ml of warm 1× HBSS with 25 mM HEPES, 4.2 mM NaHCO_3_, 1.26 mM CaCl_2_ (Sigma-Aldrich), 490 μM MgCl_2_ (Sigma-Aldrich), 406 μM MgSO_4_ (Sigma-Aldrich), and 50 mg of Collagenase Type IV (Worthington) was perfused through the liver for 15 min to disrupt the extracellular matrix. Tissue was then mechanically disrupted and passed through a 100-μM cell strainer. Hepatocytes were pelleted using centrifugation at 70g for 3 min, and non-parenchymal cells (NPCs) in the supernatant were transferred to a fresh tube. After a second hepatocyte-removal step, the NPCs were pelleted at 300g centrifugation for 5 min. RBCs were lysed by incubation in 2 ml of ACK buffer (Sigma-Aldrich) for 2 min at room temperature. NPCs underwent a 25:50 Percoll gradient to isolate liver-resident immune cells for downstream analyses.

### Flow Cytometry

An aliquot of isolated liver-resident immune cells was incubated at room temperature with mouse anti-rat Fc block (BD Biosciences), followed by fluorochrome-labeled monoclonal antibodies (**Supplementary Table S1**). Data were acquired on an Aurora spectral flow cytometer (Cytek, Bethesda, MD, USA) calibrated according to the manufacturer’s recommendations using appropriate controls. Data were analyzed in FCS Express 7 (DeNovo software, Pasadena, CA, USA).

### Quantitative Reverse Transcription Polymerase Chain Reaction

An aliquot of isolated liver-resident immune cells was immediately lysed using the RNeasy Mini Kit (Qiagen, Valencia, CA, USA) RLT buffer; mRNA was isolated following the manufacturer’s protocol. Reverse transcription was performed using an iScript cDNA synthesis kit (Bio-Rad) according to the manufacturer’s instructions. The NCBI primer design tool was used to identify accurate forward and reverse primers, which were then synthesized as 20–25-base pair DNA oligonucleotides (Integrated DNA Technologies, Newark, NJ, USA; **Supplementary Table S2**). Quantitative reverse transcription polymerase chain reaction (qRT-PCR) was performed using an Applied Biosystems 7500 fast detection system (Applied Biosystems, Foster City, CA). Total cDNA was amplified in 20 µl of PCR mix containing 250 nM of each primer, 1× SYBR Green Supermix (Bio-Rad), 5 ng cDNA, and nuclease-free water (Ambion, Inc., Austin, TX, USA) for 40 cycles. Gene expression was interpreted relative to the glyceraldehyde 3-phosphate dehydrogenase housekeeping gene using the 2^-ΔCt^ method.

### Mixed Lymphocyte Reactions

Freshly isolated lymph node-derived lymphocytes from naive Brown Norway rats were collected as responder cells for MLRs. Cryopreserved liver-resident immune cells, isolated from the livers of control and experimental Lewis rats, were recovered to serve as allogeneic stimulator cells. In Phosphate buffered saline (PBS), at a concentration of 1 × 10^6^ cells/ml, stimulators were labeled with a 1:1,000 dilution of Cell Trace Violet (CTV), while responders were separately labeled with a 1:1,000 dilution of Cell Trace Far Red (CTFR) (Thermo Fisher Scientific, Waltham, MA, USA). Stimulator cells then received 20 grays of irradiation prior to culture. In a 96-well plate, 2 × 10^5^ responders were plated with 0.5 × 10^5^ irradiated stimulators in RPMI media supplemented with 10% fetal bovine serum (FBS). Negative controls were established using irradiated CTFR-labeled leukocytes derived from naive Brown Norway livers. Non-specific stimulation using activating plate-bound anti-CD3 (1ug/mL; BD Biosciences) and soluble anti-CD28 (1 µg/ml; BD Biosciences) served as positive controls. All experimental conditions were performed in triplicate. After 4 days of culture at 37°C, cells were collected and flow cytometry data were acquired using an LSR II FACS machine (BD Biosciences). Data analysis was performed using FlowJo software (Tree Star, San Carlos, CA, USA).

### Statistical Analysis

Statistical analyses were performed in GraphPad Prism v.8.3.1. Dunnett’s multiple comparisons test or two-tailed t-tests were used as appropriate unless otherwise indicated. A p value <0.05 was considered significant.

## Results

### Liver Function During Normothermic *Ex Vivo* Liver Perfusion

**Figure 2:**
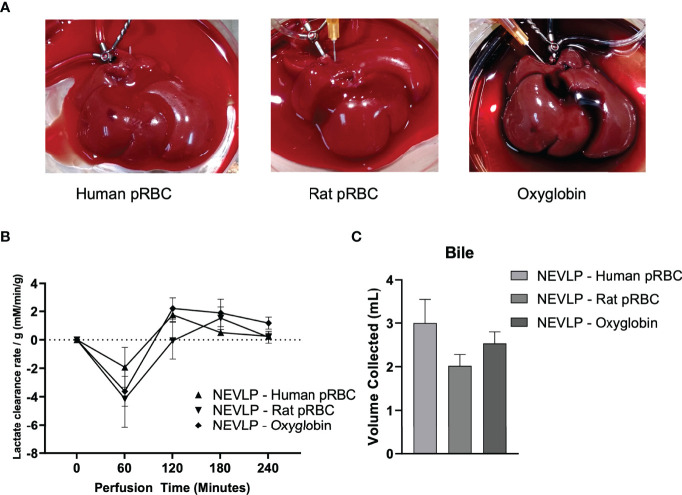
Normothermic machine liver preservation. Livers in the normothermic ex vivo liver perfusion arms underwent 4 h of normothermic preservation with human pRBC, rat packed red blood cells, or Oxyglobin as the oxygen carrier. Oxygen saturation of the perfusate was maintained at >95% throughout the duration of each perfusion, and no significance was found in portal vein pressure between groups (data not shown). Perfusate samples were collected once hourly for assessment of lactate, liver damage, and cytokine concentrations. **(A)** Representative livers undergoing perfusion from each treatment group. **(B)** Lactate clearance of livers undergoing perfusion with different oxygen carriers. **(C)** Bile was collected from the bile duct during perfusion. Due to technical failures with bile duct cannulation (2 in the human pRBC, 1 in the rat pRBC, and 2 in the Oxyglobin groups), bile production data consist of n = 4 for human pRBC and n = 6 for rat pRBC and Oxyglobin groups. Data are shown as mean ± SEM. pRBC, packed red blood cells; NEVLP, normothermic ex vivo liver perfusion.

Liver perfusion quality and liver function were assessed throughout each NEVLP experiment. All NEVLP livers demonstrated uniform perfusion and were free of gross ischemia (**Figure 2A**). Perfusate lactate concentrations were measured hourly, and the rate of lactate clearance was calculated between each subsequent hour of perfusion. There were no significant differences in lactate clearance rate observed between the groups (**Figure 2B**). Total bile production was measured at the conclusion of most experiments (**Figure 2C**), with no significant differences observed between groups.

### Liver Injury and Cytokine Production During Normothermic *Ex Vivo* Liver Perfusion

**Figure 3:**
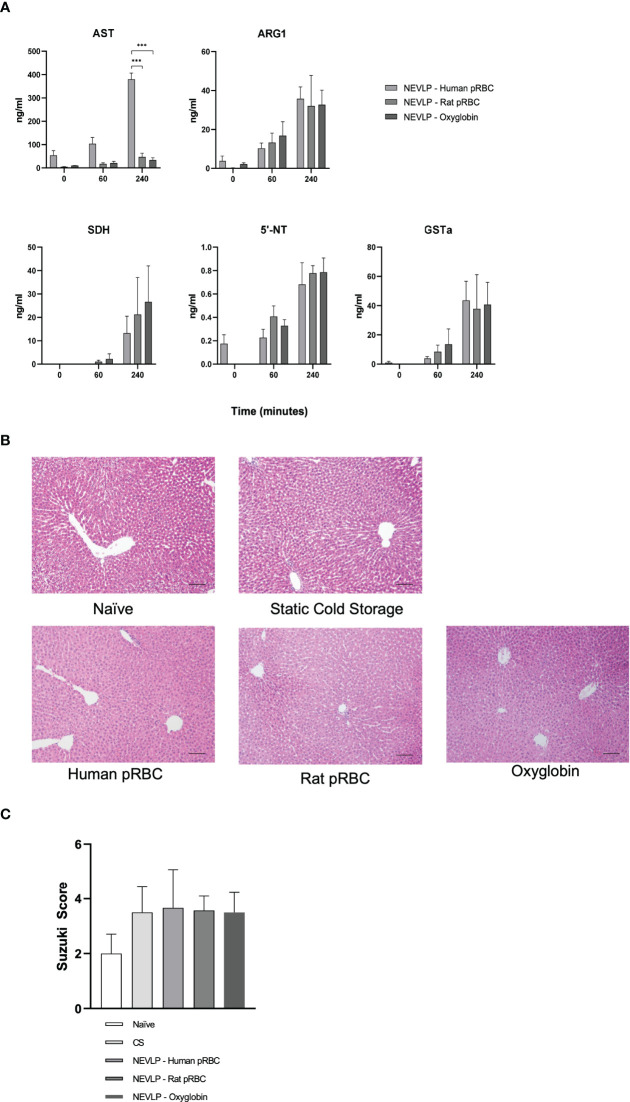
Oxygen carriers have no effect on liver damage during NEVLP. Liver injury was assessed by the presence of liver damage-associated enzymes in the perfusate and histology. **(A)** Markers of liver damage were measured in the perfusate hourly and at the conclusion of NEVLP. These experiments showed elevations in AST in the human RBC oxygen carrier group. Perfusate samples from one human pRBC and one rat pRBC NEVLP experiment were mislabeled, and timing could not be confirmed. Therefore, these samples were omitted from perfusate analysis (data consist of n = 5 for human pRBC, n = 3 for rat pRBC, and n = 5 for the Oxyglobin group). **(B)** Representative micrographs of livers stained with H&E for each treatment group. **(C)** Quantitative analysis of liver damage using the Suzuki criteria. No significant differences were found between treatment groups, and overall levels of damage were low. ***p < 0.001.

To evaluate liver injury caused by NEVLP, we analyzed the perfusate samples for markers of liver damage. Levels of ARG1, SDH, 5′-NT, and GSTα, markers of tissue injury, did not reveal any significant differences between groups, although all trended higher over the course of perfusion. In contrast, AST levels in the human pRBC group trended higher than the other groups at 0 and 60 min and were significantly higher at 240 min (p < 0.001; **Figure 3A**).

To evaluate the damage to the liver caused by NEVLP using different oxygen carriers, we performed a histopathological analysis of liver architectural damage following the Suzuki criteria. Representative H&E images demonstrate similar findings between all groups, showing that oxygen carriers during machine perfusion had little effect on liver architecture damage (**Figures 3B, C** **)**.

To determine the cytokine production capacity of liver-resident immune cells during NEVLP, we assessed the perfusate for various immune-related cytokines. During NEVLP, samples were collected at 0, 60, and 240 min of perfusion, and cytokines were evaluated with a multiplex ELISA using the cell-free perfusate supernatants. There were no significant changes in pro-inflammatory or anti-inflammatory cytokines during the perfusion between different oxygen carriers (**Supplementary Figure S1**).

### Quantitative Reverse Transcription Polymerase Chain Reaction Indicates Increased Pro-Inflammatory Cytokine Production in Human pRBC-Based Perfusates

**Figure 4:**
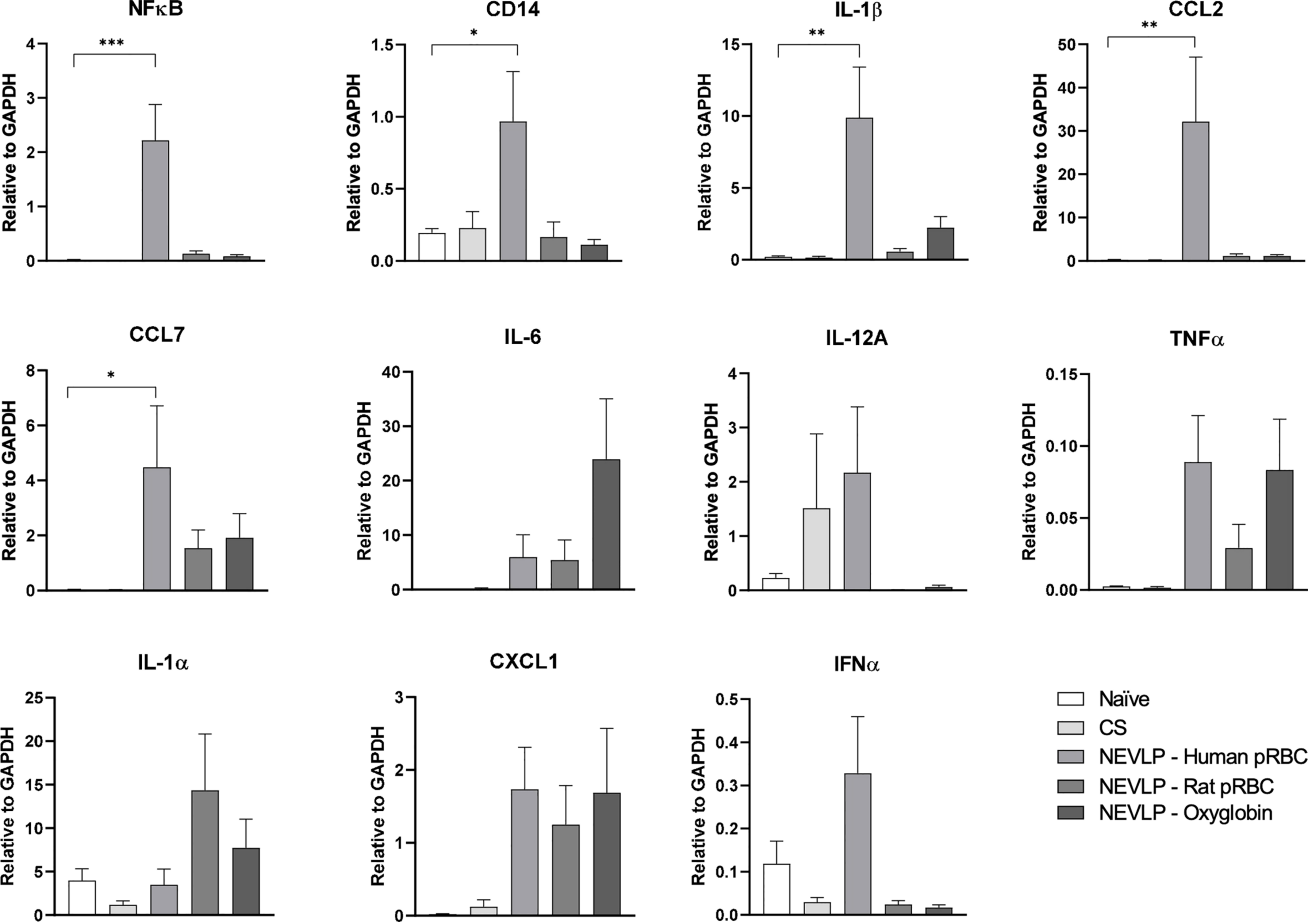
Inflammatory markers are elevated when using human pRBC for NEVLP. qRT-PCR was used to measure the gene expression of inflammatory and anti-inflammatory markers in NPCs. There were no significant differences in the majority of the markers assessed; however, the levels of IL-1, CCL2, CCL7, CD14, and NF-κB were higher in the human pRBC compared to those in naive. One human pRBC and one rat pRBC NEVLP experiment had poor cell yield after liver digestion and NPCs were prioritized for flow cytometry; therefore, data consist of naive (n = 4), SCS (n = 4), NEVLP with human pRBC (n = 5), NEVLP with rat pRBC (n = 6), and NEVLP with Oxyglobin (n = 8). *p < 0.05, **p < 0.01, ***p < 0.001.

Gene expression levels of IL-1β, CCL2, CD14, NF-κB, IL-1α, IL-6, IL-12a, TNF-α, CCL7, CXCL1, and IFN-α were measured in isolated liver NPCs by qRT-PCR. NF-κB and inflammatory markers IL-1β, CCL2, CCL7, and CD14 were significantly upregulated in the human pRBC NEVLP group compared to those in naive liver, whereas other groups were indistinguishable from naive (**Figure 4**). IL-1α, IL-6, IL-12a, TNF-α, CXCL1, and IFN-α expression levels were not significantly different between groups.

### CD86 Expression Is Higher in Human pRBC-Based Perfusate by Flow Cytometry

**Figure 5:**
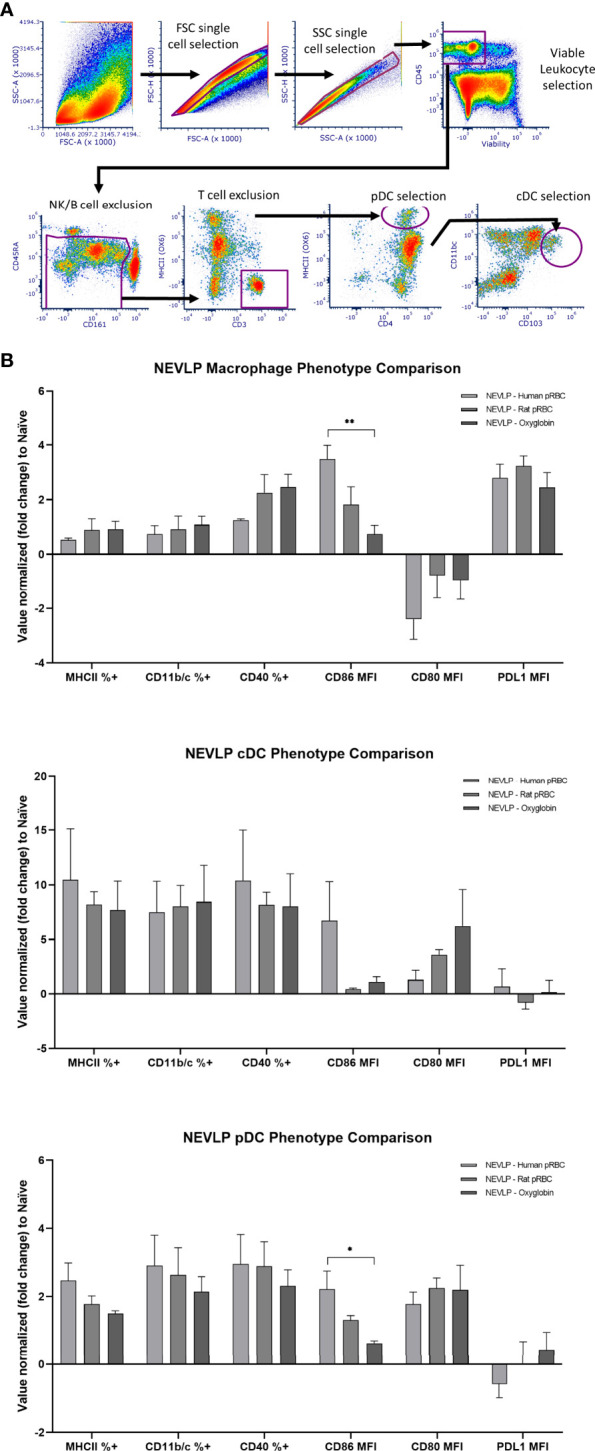
CD86 expression is higher on macrophages and pDCs after NEVLP perfused with human pRBCs. **(A)** Liver-resident immune cells were identified in the NPC compartment using the following gating strategy: after single cells and live cells were identified, immune cell identification with CD45, T-cell and B-cell exclusion using CD3 and CD45RA, and NK cell exclusion of CD161hi/MHC II- cells were done. cDCs (CD45+, CD103+, CD11bc+), pDCs (CD45+, CD4+, MHCII+, CD103-, CD11bc-), and macrophages (CD45+, CD4+, MHCII+, CD103lo, CD11bc+) were then identified as shown. **(B)** Populations were gated into positive vs. negative phenotypes when appropriate. Median fluorescence intensity (MFI) was used for signals showing variability on a gradient. Data consist of naive (n = 4), SCS (n = 4), NEVLP with human pRBC (n = 6), NEVLP with rat pRBC (n = 5), and NEVLP with Oxyglobin (n = 7). *p< 0.05, **p < 0.01.

Isolated liver NPCs were analyzed using flow cytometry using a gating strategy we recently described to identify liver-resident classical DCs (cDCs), plasmacytoid DCs (pDCs), and macrophages (22). CD86 surface expression on macrophages and pDCs was significantly higher in the rat pRBC NEVLP group compared to that in the Oxyglobin group (p < 0.01 and p < 0.05, respectively). MHCII, CD11b/c, CD40, CD80, and PD-L1 expression levels were different from those in naive samples; when comparing between each of the oxygen carrier groups, none reached statistical significance (**Figure 5**).

### Decreased Allogeneic Stimulation in Oxyglobin-Based Perfusate

**Figure 6:**
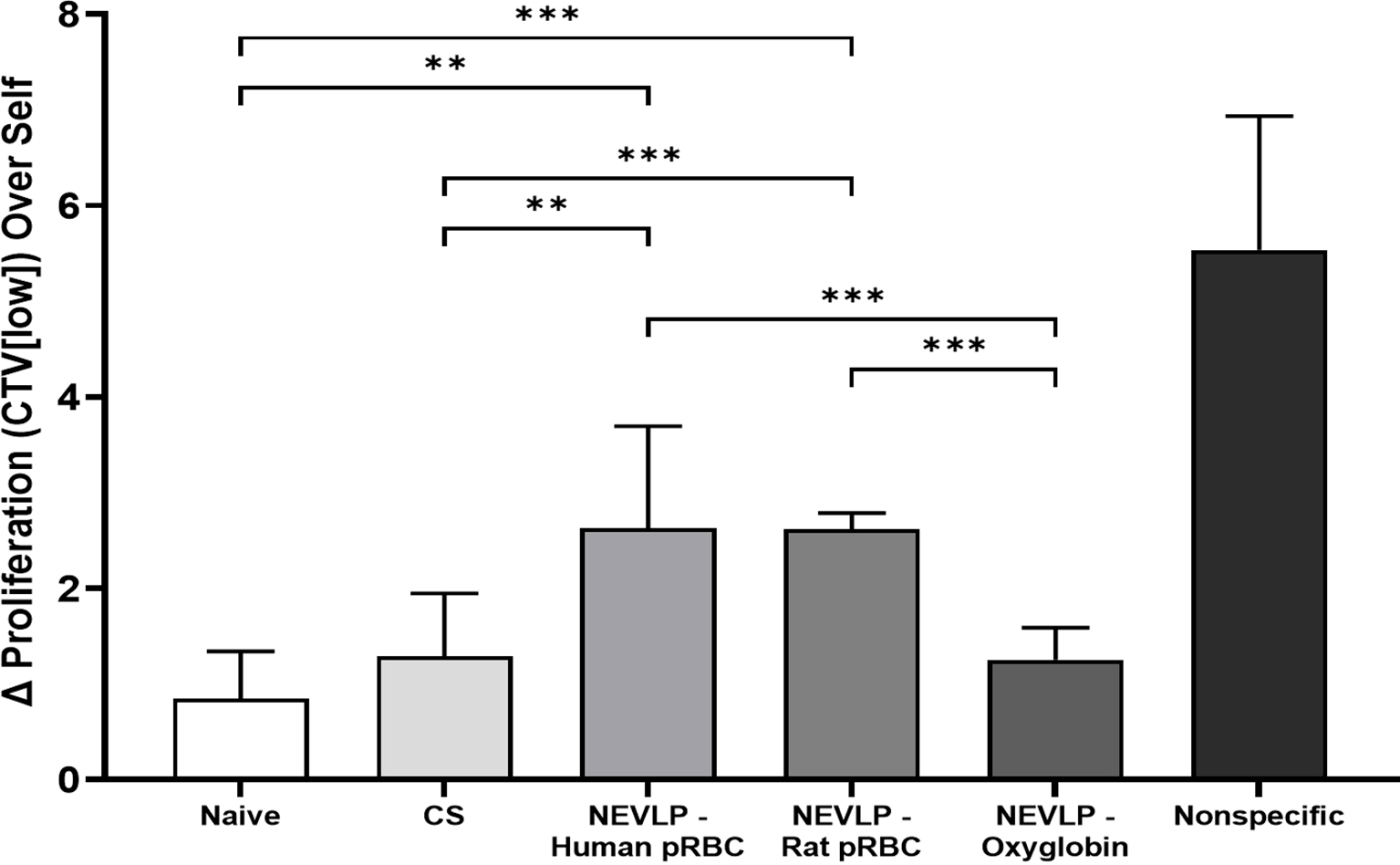
Allogeneic MLR responses are elevated after NEVLP with human pRBC or rat pRBC. Lymph node-derived Brown Norway leukocytes were cocultured with irradiated Lewis liver-resident immune cells isolated from control (naive, CS) or experimentally perfused (NEVLP-Human pRBC, NEVLP-Rat pRBC, NEVLP-Oxyglobin) livers. The MLR response was measured after 4 days in culture by the delta (Δ) increase of T-cell (CD3+) proliferation over an autologous (Brown Norway liver-resident immune cell stimulator) internal control. Statistical comparisons between allogeneic conditions (Lewis-stimulated) are shown (**p < 0.01, ***p < 0.001). The nonspecific positive control, stimulated with T cell-activating antibodies (anti-CD3/anti-CD28), yielded a significant (p < 0.001) increase in T-cell proliferation compared to all allogeneic conditions. Triplicate data were derived from n = 4 for Oxyglobin, n = 3 for human pRBC, and n = 2 for naive, CS, rat pRBC, and nonspecific groups.

Isolated liver NPCs were assessed for their ability to stimulate allogeneic responder cells in MLRs. Liver-resident immune cells isolated from the Oxyglobin group were the least stimulatory in MLRs when compared to either human or rat pRBCs (**Figure 6**).

## Discussion

In this study, our goal was to examine the immunological effects of common oxygen carriers used during NEVLP on the liver and liver-resident immune cells. Although prior studies have investigated the effect that different oxygen carriers have on organ function during normothermic perfusion (reviewed in Bodewes et al. (16), this is the first study to assess the effects of oxygen carriers on the tissue-resident immune cells.

We first show that using human pRBCs, rat pRBCs, or the hemoglobin-based oxygen carrier, Oxyglobin, as oxygen carriers in the perfusate, that each can maintain a functional liver during NEVLP. This is demonstrated by the lactate clearance and bile production of the livers during perfusion (**Figure 2**). Although the enzyme markers of liver damage increased throughout perfusion, in accordance with prior studies (20, 21), these increases were mostly independent of the oxygen carrier as shown by multiplex ELISA (**Figure 3**). The notable exception to this trend was AST in the human pRBC group, which increased rapidly and significantly throughout NEVLP when compared to those of both rat pRBC and Oxyglobin. However, given no differences in the four other liver damage-associated analytes measured, the rise in AST may be more indicative of hemolysis of the human pRBCs as opposed to liver damage (23). Although the human pRBCs were used within the expiration date, preanalytical variability in human donors, such as age and storage time (24), may have made these cells susceptible to breakdown with subsequent release of AST. However, prior studies have shown that rat RBCs show decreased deformability and membrane rigidity when compared to human RBCs, which may make them more susceptible to hemolysis (25). Nevertheless, all oxygen carrier groups showed low levels of tissue damage histologically, further supporting that RBC-based and hemoglobin-based oxygen carriers can be used in NEVLP (16, 26).

In the current study, there were no significant variations in pro-inflammatory or anti-inflammatory cytokines produced by the liver during NEVLP between the different oxygen carriers (**Supplementary Figure S1**). However, there were significant differences in the gene expression levels of certain inflammatory genes based on the oxygen carrier present during NEVLP. Specifically, in the human pRBC group, there were significantly upregulated levels of CD14, CCL2, NF-κB, and IL-1β when compared to naive liver, indicating Toll-like receptor signaling and inflammasome priming (27) (**Figure 4**). This inflammation associated with human pRBC as a perfusate component is consistent with previous reports of DAMPs released during machine perfusion (20). In contrast, inflammatory gene expression levels in the syngeneic rat pRBCs and Oxyglobin groups were not significantly different from those in naive livers.

Given the importance of liver-resident immune cells on transplantation rejection (18, 19), we sought to assess how oxygen carriers in the NVELP perfusate affect their phenotype and stimulatory function. Using spectral flow cytometric analysis of liver-resident immune cells, we demonstrated that NEVLP with human pRBCs led to a significant increase in CD86 expression on both macrophages and pDCs when compared to that in the Oxyglobin group (**Figure 5**). A higher expression of this costimulatory protein indicates that the liver-resident immune cells were activated in this group (28). These data suggest a less activated immune phenotype on liver-resident immune cells when rat pRBCs or Oxyglobin is used as an oxygen carrier. The activated immunophenotype of liver-resident macrophages and pDCs correlated with the stimulatory function seen in the MLRs with allogeneic responder cells. In these experiments, Oxyglobin showed no significant differences in stimulation over the CS or naive groups, while the human and rat pRBC groups demonstrated significantly higher stimulation indices (**Figure 6**).

Normothermic perfusion can create an inflammatory environment within the organ with subsequent increase in DAMPs and activation of tissue-resident immune cells (20, 21, 29, 30). These activated immune cells can have negative downstream consequences, and mitigating their inflammatory potential is of major clinical interest. For example, Noda et al. (30) reported using a leukocyte filter to remove these activated lymphocytes to improve lung function after normothermic preservation, and Scheuermann et al. (20) found that sub-normothermic temperatures could decrease inflammation markers. In our prior study, we added anti-inflammatory cytokines to the NEVLP circuit to keep liver-resident immune cells in a quiescent state. However, the effects of oxygen carriers on the inflammation observed in normothermic perfusion have not been fully elucidated.

One of the most common normothermic preservation oxygen carriers is pRBCs (12). However, there are potential issues with pRBCs as oxygen carriers, including immune-related phenomena and transmission of blood-borne infections (14, 31). In addition, RBCs can undergo loss of structural integrity over the course of machine perfusion, resulting in hemolysis and the release of free hemoglobin (13). Extracellular heme is a DAMP that engages inflammasome activation as a key mediator of inflammation in macrophages (32) and sustain a cycle of oxidative stress (33). Therefore, hemolysis of human pRBCs in the current study could explain the higher levels of CD14, CCL2, NF-κB, and IL-1β. However, as mentioned above, rat pRBCs may be more prone to hemolysis (25), yet there were no significant differences in the levels of any inflammatory markers in the liver-resident immune cells in this NEVLP group. Although this study did not identify the mechanism of increased inflammation in the human pRBC group, it is worth noting the effects that the oxygen carrier can have on subsequent immunological analysis.

Acellular hemoglobin-based oxygen carriers have been found to be an acceptable alternative to pRBCs for *ex vivo* organ perfusion (16). In this study, the hemoglobin-based oxygen carrier, Oxyglobin, was associated with the least amount of immune activation on liver-resident immune cells during NEVLP. As such, this study provides further support for the use of Oxyglobin as an oxygen carrier for use in NEVLP. However, hemoglobin-based oxygen carriers have been associated with vasoconstriction and the formation of methemoglobin during longer perfusions. Therefore, the decreased immune activation with Oxyglobin must be balanced with the limitations of this oxygen carrier in NEVLP (34).

Limitations of this study include the relatively short perfusion time, which was sufficient to observe differences in gene expression signatures and immune cell activation but not long enough to observe differences in cytokine production between oxygen carrier groups. Indeed, our perfusion time of 240 min is much shorter than the average clinical machine perfusion time of 548 min (7). Longer preservation times in a large animal and human model will be required in future studies to determine subtle differences in liver cytokine production. Another limitation is that although we demonstrated phenotypic and functional differences in liver-resident immune cells between oxygen carrier groups, we did not assess the relevance of these findings *in vivo*. Future studies using an allogeneic transplant model are required to investigate the *in vivo* effects of activated liver-resident immune cells following NEVLP.

In conclusion, we demonstrate that human pRBC, rat pRBC, and Oxyglobin are suitable oxygen carriers for use in NEVLP; however, Oxyglobin is associated with less liver-resident immune activation.

## Data Availability Statement

The original contributions presented in the study are included in the article/supplementary files, further inquiries can be directed to the corresponding author.

## Ethics Statement

The animal study was reviewed and approved by the Institutional Animal Care and Use Committee at UW-Madison.

## Author Contributions

HJ: Research design, performance of the research, data analysis, and writing the paper; KC: Research design, performance of research, and data analysis; CL: Performance of research, data analysis, and writing the paper; JV: Performance of research; JN: Data analysis and writing the paper; YL: Performance of the research; BV: Performance of the research; WZ: Performance of the research SM: Performance of the research and data analysis PC: Performance of the research, data analysis, and writing the paper; DA-A: Research design, performance of the research, data analysis, and writing the paper. All authors contributed to the article and approved the submitted version.

## Funding

This research was supported by the National Institute of Health (NIH) K08AI155816 awarded to DA.

## Conflict of Interest

The authors declare that the research was conducted in the absence of any commercial or financial relationships that could be construed as a potential conflict of interest.

## Publisher’s Note

All claims expressed in this article are solely those of the authors and do not necessarily represent those of their affiliated organizations, or those of the publisher, the editors and the reviewers. Any product that may be evaluated in this article, or claim that may be made by its manufacturer, is not guaranteed or endorsed by the publisher.
