# Supplementary figures and images for: The Immunological Effect of Oxygen Carriers on Normothermic Ex Vivo Liver Perfusion
Source: Front Immunol. 2022 Jun 22;13:833243. doi: 10.3389/fimmu.2022.833243 (PMC9258194; doi:10.3389/fimmu.2022.833243)

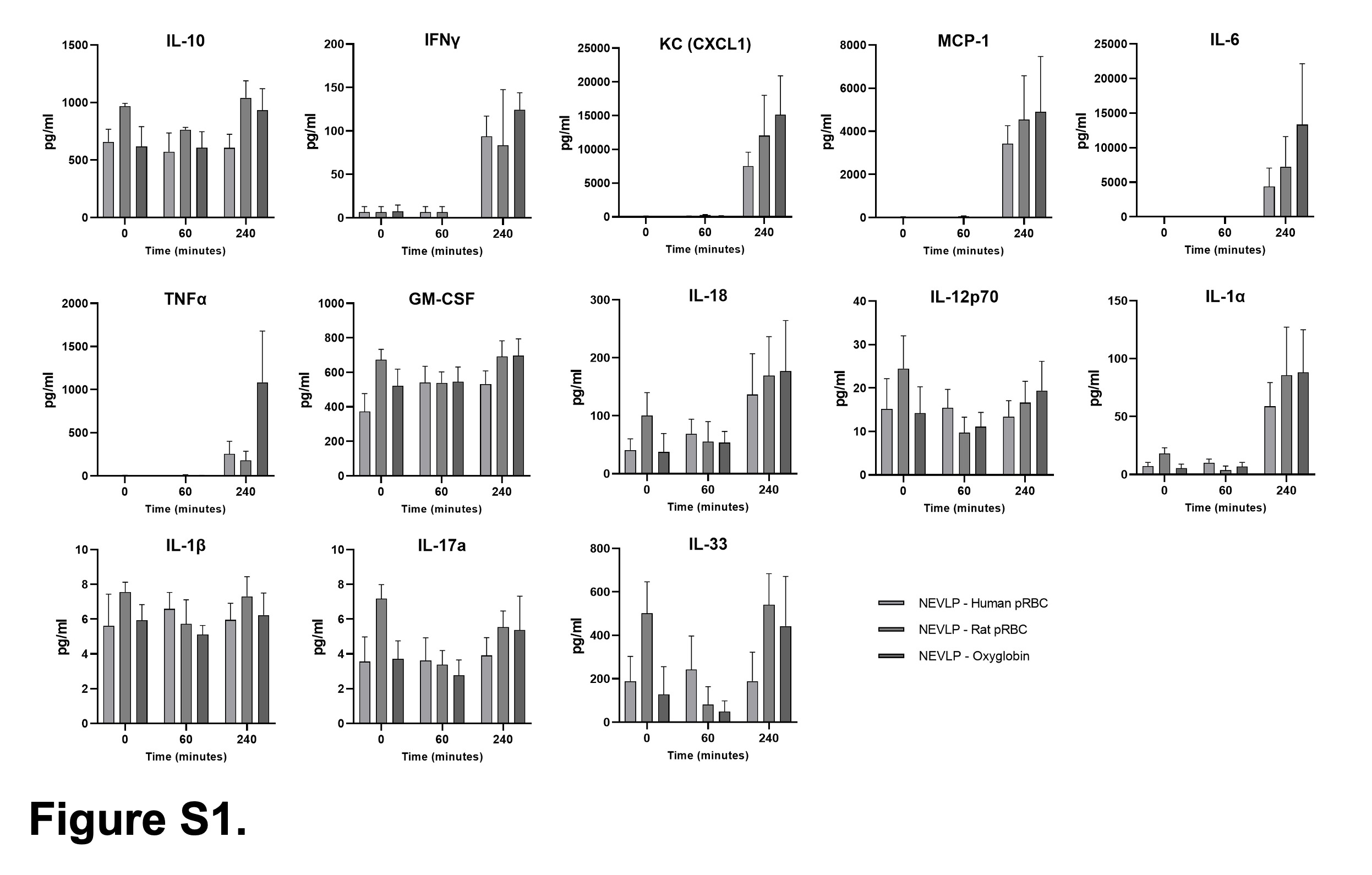

Supplement: Supplementary file 1 [file Image_1.jpeg]
