# Supplementary material for: The Immunological Effect of Oxygen Carriers on Normothermic Ex Vivo Liver Perfusion
Source: Front Immunol. 2022 Jun 22;13:833243. doi: 10.3389/fimmu.2022.833243 (PMC9258194; doi:10.3389/fimmu.2022.833243)
Supplement: Supplementary file 2 [file Table_1.docx]

| **Target** | **Fluorophore** | **Clone** | **Company** | **Catalog Number** | **Dilution** |
| --- | --- | --- | --- | --- | --- |
| Free Amines (Viability Dye) | Ghost Dye UV450 | N/A | Tonbo Biosciences | 13-0868-T100 | 1:200 |
| CD3 | BV421 | 1F4 | BD | 563948 | 1:40 |
| CD45RA | BV421 | OX-33 | BD | 740043 | 1:100 |
| RT1B (MHC II) | BV605 | OX-6 | BD | 744128 | 1:100 |
| CD11b/c | BV711 | OX-42 | BD | 743981 | 1:100 |
| Integrin αE2 (CD103) | BV786 | OX-62 | BD | 744679 | 1:100 |
| CD40 | PE | 01 | Sino Biological | 80151-MM01-P | 1:100 |
| PD-L1 | Conjugated in-house to CF594 (Biotium) | 2B11D11 | ProteinTech | 66248-Ig | 1:20 |
| CD161 | PE-Cy7 | 3.2.3 | Biolegend | 205610 | 1:100 |
| CD4 | FITC | W3/25 | Biolegend | 201505 | 1:100 |
| RT1A (MHC I) | FITC | OX-18 | BD | 554919 | 1:40 |
| CD86 | BB700 | 24F | BD | 746002 | 1:40 |
| CD45 | A647 | OX-1 | Biolegend | 202212 | 1:100 |
| CD80 | APC | 3H5 | Invitrogen | MR6505 | 1:20 |
| CD4 | APC-Cy7 | W3/25 | Biolegend | 201518 | 1:100 |
| Free Amines (Viability Dye) | Ghost Dye Red 780 | N/A | Tonbo Biosciences | 13-0865-T100 | 1:200 |

**Table S1. Antibodies used for flow cytometry.** All antibodies were titrated as single colors at concentrations of 1:100, 1:40, 1:20, and 1:10 prior to use in the multi-color panel. FMO controls were used for gating and data analysis.
