# Supplementary material for: The Immunological Effect of Oxygen Carriers on Normothermic Ex Vivo Liver Perfusion
Source: Front Immunol. 2022 Jun 22;13:833243. doi: 10.3389/fimmu.2022.833243 (PMC9258194; doi:10.3389/fimmu.2022.833243)
Supplement: Supplementary file 3 [file Table_2.docx]

| **Target** | **F/R** | **Primer Sequence (5’ to 3’)** |
| --- | --- | --- |
| GAPDH | Forward | CTGCACACTCGCTTCCTAGAG |
| GAPDH | Reverse | AGGAAGGCCTTGACCTTTTCAG |
| IL-1α | Forward | CGCTTGAGTCGGCAAAGAAATC |
| IL-1α | Reverse | TCAGAGACAGATGGTCAATGGCA |
| IL-1β | Forward | CCTCTCCAGTCAGGCTTCCTT |
| IL-1β | Reverse | GGTCATTCTCCTCACTGTCGA |
| IL-6 | Forward | CTTGGAAATGAGAAAAGAGTTGTGC |
| IL-6 | Reverse | ACGGAACTCCAGAAGACCAGA |
| IL-12A | Forward | ATGTGTCAATCACGCTACCTCCTC |
| IL-12A | Reverse | TCGATGTCTCCAGCAGTGCAA |
| TNFα | Forward | TCGGTCCCAACAAGGAGGAG |
| TNFα | Reverse | GCTTGGTGGTTTGCTACGAC |
| CCL2 | Forward | ATGCAGGTCTCTGTCACGCT |
| CCL2 | Reverse | GGGCATTAACTGCATCTGGC |
| CCL7 | Forward | GCACCGAGTCTGCCAACTTTC |
| CCL7 | Reverse | GTGGATGAATTGGTCCCATCTGGTT |
| CXCL1 | Forward | CACCCAAACCGAAGTCATAGCC |
| CXCL1 | Reverse | GAAGCCAGCGTTCACCAGAC |
| IFNα | Forward | GAAGACTCCCTACTGGCTGT |
| IFNα | Reverse | ATTTCTTTCTTCTCTCAGTCTTCCC |
| CD14 | Forward | CGGATATTCTGGCCTCCGGG |
| CD14 | Reverse | TGTTGAGATCGGGTCCGGTG |
| NFKB1 | Forward | TCGGAACTGGGCAAATGTTTCA |
| NFKB1 | Reverse | GCACACGTAGCGGAATCGAA |

**Table S2. Primers used for qRT-PCR.** Primers were designed using NCBI primer design tools to ensure target specificity and avoid genomic DNA amplification. Melt curves and gel electrophoresis were performed to assess primer performance.
